# The relationship of parenting style and eating behavior in preschool children

**DOI:** 10.1186/s40359-022-00981-8

**Published:** 2022-11-22

**Authors:** Anaëlle L. Leuba, Andrea H. Meyer, Tanja H. Kakebeeke, Kerstin Stülb, Amar Arhab, Annina E. Zysset, Claudia S. Leeger-Aschmann, Einat A. Schmutz, Susi Kriemler, Oskar G. Jenni, Jardena J. Puder, Simone Munsch, Nadine Messerli-Bürgy

**Affiliations:** 1 Department of Psychology, University of Fribourg, Rue P.A. de Faucigny 2, 1700 Fribourg, Switzerland; 2 Institute of Psychology, FADO, University of Lausanne, Géopolis, 1015 Lausanne, Switzerland; 3 Department of Clinical Psychology and Psychotherapy, University of Fribourg, Rue P.A. de Faucigny 2, 1700 Fribourg, Switzerland; 4 Department for Psychology, University of Basel, Missionsstrasse 62A, 4055 Basel, Switzerland; 5 Child Development Center, University Children’s Hospital Zurich, Steinwiesstrasse 75, 8032 Zurich, Switzerland; 6 Children’s Research Center, University Children’s Hospital Zurich, Steinwiesstrasse 75, 8032 Zurich, Switzerland; 7 Obstetric Service, Department Women-Mother-Child, Lausanne University Hospital, Lausanne, Switzerland; 8 Epidemiology, Biostatistics and Prevention Institute, University of Zurich, Hirschengraben 84, 8001 Zurich, Switzerland

**Keywords:** Eating behavior, Preschool, Parenting style, SPLASHY

## Abstract

**Background:**

Eating behavior represents individual appetitive traits which are related to the individual’s regulation of food intake. Eating behavior develops at an early age. There is some evidence that parenting styles might impact on the child’s eating behavior. The aim of this study was to investigate the relationship of different dimensions of positive and negative parenting styles with the child’s eating behavior at a critical age period of the child’s early development.

**Methods:**

Parents of 511 preschool children (aged 2–6 years) completed the Children Eating Behavior Questionnaire and the Alabama Parenting Questionnaire.

**Results:**

Analyses revealed that different dimensions of negative parenting styles were associated with eating behavior of the child. In details, inconsistent parenting showed a consistent association with eating behavior of a child (i.e. higher emotional eating, higher food responsiveness, higher food fussiness, higher satiety responsiveness and more enjoyment of food), whereas corporal punishment was associated with more emotional overeating and more food responsiveness but less satiety responsiveness. Further, powerful implementation was related to higher food responsiveness and less enjoyment of food and low monitoring was associated with higher emotional overeating and more slowness in eating. There was no such consistent association of positive parenting and eating behavior.

**Conclusions:**

More negative parenting styles were associated with eating behavior which is more often related to potential weight problems in a long term, whereas positive parenting did not show such a consistent relationship with eating behavior. Negative parenting should be in the focus of prevention and treatment of eating behavior problems in young children.

*Trial registration*: ISRCTN41045021 (06/05/2014).

## Background

Eating behavior represents individual appetitive traits which are related to the person’s regulation of food intake [1]. Eating behavior develops already in early childhood and individual differences in appetitive and satiety traits are therefore determined early in a child’s life [2–4]. Especially the preschool age is a critical age period for its development. Previous research has shown that the eating behavior of a child at preschool age remains stable over childhood and up to adulthood [5, 6], and influences the child’s growth and weight [7]. Therefore, problems in eating behavior at preschool age are related to increased eating- and weight-related problems at a later age and may cause long-term health consequences in adolescents and adults [4, 6–10].

Eating behavior is divided into food approaching and food avoidant behavior. Food approaching behavior includes behavior which involves increased food intake (e.g. high emotional overeating, high food responsiveness and increased food enjoyment when eating). Food avoidant behavior represents restrictive and selective eating behavior which correspond to less food intake such as emotional undereating and picky eating or to the child’s ability to reduce food intake after eating such as high satiety responsiveness [11]. Food approaching and food avoidant behaviors may both be related to negative weight consequences in early childhood. Whereas approaching eating behavior is related to an increased risk for overweight and obesity on a long term, avoidant behavior has been associated with future problems of underweight [5, 7, 11–15].

The development of a child’s eating behavior at preschool age is influenced by the parental behavior at meal and snack times and by the child’s disposition (i.e. satiety responsiveness [16]). There is evidence that parents not only contribute to the child’s development of food preferences [17] but their parental feeding practices shape the child’s beliefs and attitudes towards food [16] and determine the child’s eating behavior in the long run [3, 4, 16, 18]. Previous research has shown that feeding practices such as encouraging and rewarding practices increase food approaching behavior and might even reduce satiety responsiveness in children [19–21]. Other feeding practices (e.g. pressuring to eat) rather reduce food intake and limit food enjoyment [3].

Besides feeding practices, parenting styles which are not specifically related to feeding but rather represent the general communication attitudes of parents towards their child [22, 23] might contribute to the complex situation of daily food intake in a family’s life. Parenting styles might influence the general environmental conditions of a family’s mealtimes and impact on specific eating behavior such as emotional over and undereating but also other dimensions of child’s eating behavior (e.g. food enjoyment). However, research on the impact of parenting styles on eating behavior in preschoolers has been little investigated so far. A systematic review identified seven studies on this topic [24] and revealed a weak to moderate relation between parenting style and feeding practices [24] which might indicate that these are two different aspects of parental behavior towards a child. In line with this idea, parenting style has been found to moderate the impact of feeding practices on the child’s eating behavior [25] and therefore may also contribute to problematic eating behavior on its own [26].

Parenting style is a parental trait to communicate with the child and aims at influencing the child’s behavior [27]. Parenting style is categorized at the origin by the dimensions of demandingness (parents provide limits and assure structure to control and monitor the child’s behavior) and responsiveness (parents provide warmth and understanding according to the needs of a child to develop autonomy) [22, 26]. A combination of high levels of demandingness and high levels of responsiveness is defined as a positive parenting style [28–30] which is considered the most beneficial in Western countries [22, 31, 32]. Positive parenting is associated with healthier parental feeding of preschoolers [33] and predicts less behavioral problems in preschool children [22, 34, 35]. In a systematic review by Sleddens et al. [36], studies involving children up to 18 years showed that a more positive parenting predicts healthier outcomes in childhood such as more physical activities and a healthier diet (e.g., lower caloric intake). Several studies focused on the effect of positive parenting on eating behavior in children, adolescents and young adults so far. They revealed that an authoritative parenting style, a positive parenting style (e.g. combination of high responsiveness and high demandingness), is related to less food fussiness in school-aged children [37], a more healthy diet at school-age and during adolescence [38–43] and less emotional overeating in young adults [44]. Only one study included children at preschool and early school age (age range of 2.8–7.5 years) and confirmed that authoritative parenting was related to less emotional overeating and less food fussiness at that early age period [45]. However, other dimensions of eating behavior were not investigated in that study, although there is evidence that they (e.g. emotional undereating, food and satiety responsiveness or enjoyment of food) contribute to long-term eating and weight problems [5, 7, 11–15].

Besides the positive impact of positive parenting on eating behavior, there is some evidence that negative parenting styles are related to negative parental feeding and more eating behavior problems [46, 47] as well as other behavioral problems in children and preadolescents [27, 48–50]. Negative parenting is defined by a lack of warmth, of responsiveness, either a complete dismiss of control or an overcontrol on the child such as a lack of monitoring or an augmented use of strict discipline, or a complete inconsistency in responding to a child’s behavior or needs [22, 29, 51, 52]. Previous studies investigated some of these negative parenting styles in relation to eating in children. They revealed that inconsistent parenting is related to more consumption of junk food and a higher risk for eating behavior problems in a clinical sample of preschool children and even in young adults aged 19 years [46, 47]. Further, authoritarian parenting style (more controlling and using stricter disciplines) was related to more emotional overeating in the previously mentioned study with 496 preschool and early-school aged children [45], which is known to increase the risk for eating behavior problems in preschoolers [3]. Moreover, Goodman et al. [45] found that permissive parenting (e.g. dismiss of control) is associated with higher fussiness which had previously been found in a small sample of 77 English children aged 3–8 years [53]. To sum up, so far only two studies [45, 53] investigated the relation between negative parenting style and eating behavior in children at preschool age and only one study focused on the relationship of positive parenting and eating behavior. As preschool age is known to be a critical time period for the development of eating behavior, there is a need of profound knowledge to understand these relations and potentially adapt preventive and therapeutic strategies. Therefore, this study aimed to provide an overview on the relationship of different dimension of general parenting styles and of the different facets of the child’s eating behavior at preschool age and to detect the specific links between parenting style and eating behavior at that early age.

## Methods

### Study sample and design

The Swiss Preschooler’s Health Study (SPLASHY) is a multi-site prospective cohort study including 555 children within two sociocultural areas of Switzerland (German and French speaking part) (ISRCTN41045021; for details [54]). Children were recruited from 84 childcare centers within five cantons of Switzerland (Aargau, Bern, Fribourg, Vaud, Zurich) which made up 50% of the Swiss population in 2013. Recruitment was ongoing between November 2013 and October 2014 when children were 2–6 years old. Parents were asked to give their written informed consent for study participation before completing a set of questionnaires. The study was approved by all local ethical committees (No 338/13 for the Ethical Committee of the Canton of Vaud as the main ethical committee) and is in accordance with the Declaration of Helsinki. The detailed study design and the overall objectives have been previously described [54].

### Assessment

#### Child eating behavior

Eating behavior was assessed by the German and French version of the Child Eating Behavior Questionnaire (CEBQ) of Wardle and al. [56]. The questionnaire is validated in children from 2 to 9 years old [55, 56] and includes eight subscales and 35 items using a 5 point-Likert scale (never (1) to always (5)). Subscales focus on the child’s eating behavior and include *food responsiveness* (e.g., “given the choice, my child would eat most of the time”), *enjoyment of food* (e.g., “my child enjoys eating”), *emotional overeating* (e.g., “my child eats more when worried), *satiety responsiveness* (e.g., “my child gets full before his/her meal is finished”), *slowness in eating* (e.g., “my child eats slowly”), *emotional undereating* (e.g., “my child eats less when upset”), *food fussiness* (e.g., “my child refuses new food at first”) and *Desire to drink* (e.g., “my child always asks for something to drink”). Depending on the scale, very high or very low levels indicate unhealthy eating behavior, however no cut-off values exist to demonstrate behaviors that can be classified as dysfunctional. The German and French version of the CEBQ revealed a 7-factor structure solution TLI = 0.954, CFI = 0.952, RMSEA = 0.063 and SRMR = 0.067) [57] excluding the subscale desire to drink but proofing high validity and reliability in the German and French version with omega’s coefficients ranging from 0.66 (satiety responsiveness) to 0.90 (food fussiness) and the alpha’s values ranging from 0.69 (satiety responsiveness) to 0.89 (food fussiness) [57], comparable to the alpha’s values from the original version ranging from 0.72 (emotional overeating) to 0.91 (food fussiness) [56].

#### Parenting style

The parenting style was assessed using the Alabama Parenting Questionnaire (APQ: [35]). The APQ comprehends 40 items and seven subscales (for details see Table 1). *Positive parenting styles* consisted of the subscales: *Parental involvement* (e.g., “you drive your child to special activities”), *Positive parenting* (e.g., “you have a friendly talk with your child”), and *Responsible parenting* (e.g., “you explain your child how to behave in a specific situation”) which all are in line with the authoritative parenting. *Negative parenting styles* include the subscales *Powerful implementation*, which is comparable to authoritarian (e.g., “if your child negotiates with you, you’re giving clear instructions”), *Inconsistent parenting* (e.g., “you threatened to punish your child and then do not actually punish him/her”), *Corporal punishment* (e.g., “you hold your child firmly or shake him/her, if he/she did something wrong”), and *Low monitoring* (e.g., “your child is not at home and you don’t know where he/she is exactly”) which is in line with permissive parenting. High levels in the different scales representing higher frequency of parenting style in the daily life of a parent, however no cut-off values exist related to the subscales of APQ. The reliabilities to the APQ factors were ranging from 0.68 (Corporal punishment) to 0.85 (Positive parenting) for alpha values, findings that are comparable to the study among children of an elementary school with reliabilities with alpha values of 0.60 (Corporal punishment) to 0.84 (Positive parenting) [35].

**Table 1:** Descriptive statistics of factors from the Children Eating Behavior Questionnaire (CEBQ) and the Alabama Parenting Questionnaire (APQ)

Factors	N	M (SD)	Omega coefficient
**Parenting styles**			
***Positive parenting***			
Parental involvement, 4 items	511	4.2 (0.53)	.49
Positive parenting, 6 items	510	4.5 (0.38)	.76
Responsible parenting, 6 items	511	3.8 (0.53)	.68
***Negative parenting***			
Powerful implementation, 5 items	508	3.5 (0.61)	.72
Inconsistent parenting, 5 items	511	2.5 (0.54)	.69
Corporal punishment, 4 items	509	1.6 (0.55)	.59
Low monitoring, 5 items	510	1.3 (0.38)	.61
**Eating behaviors**			
Food responsiveness, 4 items	509	2.0 (0.75)	.83
Emotional overeating, 4 items	504	1.5 (0.55)	.77
Enjoyment of food, 5 items	509	3.5 (0.47)	.86
Satiety responsiveness, 4 items	511	2.9 (0.65)	.66
Slowness in eating, 4 items	509	2.9 (0.74)	.76
Emotional undereating, 3 items	507	3.0 (0.87)	.78
Food fussiness, 6 items	509	2.9 (0.79)	.90

Behaviors are rated on a five -point Likert scale. These are the factors retained for our 7-factors structure model of CEBQ and the 5 retained factors for APQ

### Statistical analyses

Statistical analyses were conducted using R [58], including the package lavaan [59]. As 44 of the 555 children provided no data on the relevant study characteristics, 511 children were included in the analysis. Descriptive statistics including the means ± SD for continuous variables and frequencies and percentages for categorical variables are reported. To analyze the impact of parenting style on eating behavior in these preschool children, structural equation models (SEM) were set up with the seven APQ subscales as predictors and the seven CEBQ subscales as outcomes. Seven different models were conducted, one for each of the following APQ subscales: parental involvement, positive parenting, responsible parenting, powerful implementation, inconsistent parenting, corporal punishment, and low monitoring. Each model contained the respective APQ subscale as predictor and all seven CEBQ subscales as outcomes. This way (a) all seven path coefficients and (b) the differences among all pairs of path coefficients could be estimated. Analyses were controlled for potential correlates with age, gender, language area, BMI of both parents and parenting stress level. Subscales of both CEBQ and APQ were not operationalized using sum scores since these pose problems with respect to validity and reliability [60]. Instead, we set up measurement models for each subscale (CEBQ or APQ) involved. Items of both questionnaires APQ and CEBQ subscales were all measured on an ordinal scale (range 0–4). Thus, the mean and covariance adjusted weighted least squares estimator (WLSMV) was used to compute model parameters and their standards errors. In order to report model fit indices, the robust versions of the Tucker Lewis Index (TLI), the root mean square error of approximation (RMSEA), and the standardized root mean square residual (SRMR) are provided by considering the required critera of an acceptable model fit for these indices: RMSEA (≤ 0.06, 90% CI ≤ 0.06, Cfit not significant), SRMR (≤ 0.08), CFI (≥ 0.95), and TLI (≥ 0.95) [61]. To estimate reliabilities of the factors specified in the measurement models, omega coefficient [62] was used, which is known to be more useful than the often-used Cronbach’s alpha [63]. No attempt was made to control for multiple testing as we considered our analyses to be of explorative nature.

## Results

### Descriptive statistics

Mean age in the sample (n = 511) was 3.85 years (SD = 0.69, ranging from 2.21 to 6.64 years) and 47% of the participants were girls. A total of 76% were living in the German-speaking part of Switzerland and 24% in the French-speaking part. Mean SES of the family was 62.88 (SD = 14.97) and higher than in the Pisa study (Swiss sample = 53.00) of OECD countries [64]. Mean age of mothers were 37.17 (SD = 4.92) and of fathers 39.86 (SD = 6.31). More than half of the children were living in rural parts of the country (59,6%) and a total of 58,7% had one or both parents being migrants. Mean levels of eating behavior subscales were comparable to the original version [56], ranging from 1.5 (*emotional overeating*) to 3.5 points (*enjoyment of food*) (see Table 1).

### Relation of parenting styles and the child’s eating behavior

Model fits of the seven SEMs (one for each of the seven APQ subscales) were all satisfactory, with robust values ranging from 0.945 to 0.951 for TLI, 0.044 to 0.049 for RMSEA, and 0.059 to 0.064 for SRMR (values for the RMSEA of the corresponding null models varied between 0.33 and 0.34). Regression analyses revealed several patterns (see Table 2 and 3). The 95 CI of the RMSEA varied between 0.42 and 0.45 for the lower limit, and between 0.49 and 0.52 for the upper limit.

**Table 2:** Regression coefficients of the relationships of positive parenting styles (predictors, in bold type) on the child’s eating behavior

Effect	Estimate	*SE*	z-value^1^	*p-*value	Standardized estimate
**Parental involvement**					
Food responsiveness	−0.050	0.067	−0.753	.0451	−0.044
Emotional overeating	−0.096	0.085	−1.129	0.259	−0.075
Enjoyment of food	−0.089	0.082	−1.076	0.282	−0.066
Satiety responsiveness	−0.054	0.084	−0.638	0.524	−0.043
Slowness in eating	0.021	0.078	0.274	0.784	0.017
Emotional undereating	−0.064	0.094	−0.677	0.498	−0.042
Food fussiness	−0.104	0.084	−1.245	0.213	−0.071
**Positive parenting**					
Food responsiveness	−0.142	0.075	−1.890	0.059	−0.111
Emotional overeating	−0.234	0.096	−2.441	0.015	−0.160*
Enjoyment of food	−0.157	0.087	−1.805	0.071	−0.103
Satiety responsiveness	0.020	0.088	0.233	0.816	0.014
Slowness in eating	0.040	0.082	0.481	0.630	0.028
Emotional undereating	0.028	0.097	0.283	0.777	0.016
Food fussiness	−0.117	0.089	−1.318	0.188	−0.070
**Responsible parenting**					
Food responsiveness	−0.014	0.077	−0.187	0.852	−0.010
Emotional overeating	−0.036	0.100	−0.360	0.719	−0.022
Enjoyment of food	−0.194	0.089	−2.169	0.030	−0.112*
Satiety responsiveness	−0.007	0.097	−0.074	0.941	−0.004
Slowness in eating	−0.078	0.091	−0.857	0.391	−0.048
Emotional undereating	0.017	0.111	0.152	0.879	0.009
Food fussiness	−0.070	0.096	−0.724	0.469	−0.037

Cohen's classification for effect sizes are .1 to be small, .3 to be medium, and .5 to be large.

*p < .05; ** *p* < .01; *** *p* < .001.

^1^Statistic for the test of regression coefficients against 0

**Table 3:** Regression coefficients of the relationships of negative parenting styles (predictors, in bold type) on the child’s eating behavior

Effect	Estimate	*SE*	z-value^1^	*p-*value	Standardized estimate
**Inconsistent parenting**					
Food responsiveness	0.149	0.055	2.692	0.007	0.165**
Emotional overeating	0.258	0.064	4.058	0.000	0.250***
Enjoyment of food	0.178	0.064	2.789	0.005	0.166**
Satiety responsiveness	0.253	0.068	3.742	0.000	0.250***
Slowness in eating	0.108	0.061	1.775	0.076	0.108
Emotional undereating	0.222	0.067	3.334	0.001	0.185**
Food fussiness	0.179	0.063	2.837	0.005	0.152**
**Corporal punishmen**					
Food responsiveness	0.262	0.075	3.489	0.000	0.246***
Emotional overeating	0.273	0.094	2.919	0.004	0.224**
Enjoyment of food	−0.019	0.085	−0.228	0.820	−0.015
Satiety responsiveness	−0.194	0.087	−2.238	0.025	−0.163*
Slowness in eating	0.041	0.082	0.504	0.614	0.035
Emotional undereating	−0.009	0.095	−0.092	0.927	−0.006
Food fussiness	−0.048	0.083	−0.581	0.561	−0.035
**Powerful implementation**					
Food responsiveness	0.146	0.058	2.492	0.013	0.132*
Emotional overeating	0.022	0.074	0.291	0.771	0.017
Enjoyment of food	−0.183	0.070	−2.602	0.009	−0.141**
Satiety responsiveness	0.013	0.073	0.184	0.854	0.011
Slowness in eating	−0.061	0.068	−0.888	0.375	−0.050
Emotional undereating	0.050	0.088	0.575	0.566	0.034
Food fussiness	−0.128	0.073	−1.737	0.082	−0.089
**Low monitoring**					
Food responsiveness	0.119	0.066	1.182	0.070	0.138
Emotional overeating	0.207	0.076	2.725	0.006	0.210**
Enjoyment of food	0.114	0.068	1.669	0.095	0.111
Satiety responsiveness	0.135	0.072	1.886	0.059	0.139
Slowness in eating	0.145	0.066	2.196	0.028	0.150*
Emotional undereating	0.084	0.079	1.058	0.290	0.072
Food fussiness	0.063	0.066	0.951	0.341	0.056

Cohen's classification for effect sizes are 0.1 to be small, 0.3 to be medium, and 0.5 to be large.

*p < .05; **p < .01; ***p < .001.

^1^Statistic for the test of regression coefficients against 0

Positive parenting styles were hardly related to any CEBQ subscale. In details, high levels of positive parenting were related to low levels of emotional overeating and further high levels of responsible parenting was related to low levels of enjoyment of food. There was no other significant relation of positive parenting or responsible parenting and eating behavior, nor did parental involvement as a positive parenting style play a role on eating behavior of the child in this sample.

In contrast, there were several negative parenting styles linked to the child’s eating behavior. First, inconsistent parenting was positively associated to most of the eating behavior subscales in these preschool children, except with slowness in eating. Thus, high levels of inconsistent parenting were associated with high levels in food responsiveness, in emotional over- and undereating, but also with high levels in enjoyment of food, in satiety responsiveness, and in food fussiness what was contrary to our expectations (see Table 3).

Secondly, high levels of corporal punishment were specifically related to more food responsiveness and more emotional eating and less satiety responsiveness in these children, whereas high levels of powerful implementation were related to more food responsiveness and less enjoyment of food. Finally, low levels of monitoring were related to more emotional overeating and more slowness in eating.

## Discussion

In a sample of 511 children, we investigated the relation between positive and negative parenting styles on children’s eating behavior during children’s preschool age. Although we expected that both parenting styles would be related to eating behavior, this was not the case. While the different negative parenting style subscales were often related to the different eating behavior subscales in children, the different positive parenting subscales were mostly not. For example, the subscale “inconsistent parenting” had the most consistent association with the different subscales of children’s eating behavior. Further negative parenting styles were related to the food approaching behaviors “food responsiveness” and “emotional overeating”.

More precisely, inconsistent parenting was associated with every eating behavior subscale, except for slowness in eating but only with small to medium effect sizes. This means, high levels of inconsistent parenting were associated with high levels of food responsiveness, emotional overeating, enjoyment of food, but also emotional undereating, food fussiness and unexpectedly with satiety responsiveness. These results reveal that inconsistent parenting (representing the extent to which parents are not able to enforce rules and consequences in a consistent way), is associated with high levels of food approaching and at the same time with high levels of food avoidant behavior which are both known to be related to weight problems, such as overweight and underweight problems in school-aged and preschool children [11, 14, 37, 65]. The role of inconsistent parenting has not been investigated in the previous studies on eating behavior in young children. Additionally, there is only one study on a clinical sample of preschool with leukemia where inconsistent parenting was related to the diet (more junk food), but eating behavior was not in the focus of that study [47]. However, inconsistent parenting has previously been found to negatively impact the child’s development. A previous meta-analysis revealed that only inconsistent parenting had a profound impact on the development of pediatric obesity [66] and none of the other parenting styles. Therefore, it can be assumed that this parenting style might play a specific role in the determination of eating and weight-related conditions of children and could be associated with the risk to use eating as a coping strategy to solve problems as shown in studies focusing on emotional eating [67, 68]. Surprisingly, higher use of inconsistent parenting was also related to higher satiety responsiveness, which is an adaptive behavior as it is limiting food intake when eating and therefore balancing energy intake and energy consumption supporting a healthy weight condition as a consequence [69, 70]. To our knowledge, no other study had investigated this relation of inconsistent parenting and satiety responsiveness so far. Therefore, a comparison of these contradictory findings with the literature is not possible. However, there are two explanations for these findings. First, as correction for multiple testing was not considered (due to the exploratory nature of the paper), the results could potentially be seen as a random effect, but coefficient level was relatively high with CE = 0.253. Another explanation would be related to the items of the questionnaire and the question to which extent parents might have misunderstood the content. In relation to satiety responsiveness, the CEBQ asks parents about finishing plates, not eating when snacking before, easily getting full up etc. which might have which might have been experienced as unsettling and therefore seen as a problematic eating behavior for parents, especially if these parents tend to be more inconsistent in parenting.  On the other hand, problematic eating behavior might also provoke distress in parents and negatively impact on their parenting style which means that the cause-effect relation of parenting styles and eating behaviors remain still unclear.

Our findings further revealed that high levels of corporal punishment were related to more food responsiveness, more emotional overeating, and less satiety responsiveness which all represent food approaching behaviors that are associated with more eating and weight problems on a long term [5, 7, 11–15]. To our knowledge, this is the first study which investigated the role of corporal punishment on eating behavior in preschool children. We assume that corporal punishment might provoke a more externally or cue-driven eating behavior which is related to more eating behavior problems such as more food responsiveness, higher food intake and less satiety and might represent a coping strategy too. These parenting styles might provoke more negative emotions such as fear or anger in the parent–child interaction which demands emotion regulation strategies, that are potentially not available at preschool age and might be replaced by increased food intake in such stressful conditions (emotional overeating) [71].

Finally, powerful implementation, which is related to the authoritarian style [35], was associated with more food responsiveness and less enjoyment of food in these preschool children. This result is in line with another study. Van Der Horst and Sleddens [72] found similar results in toddlers. Authoritarian parenting style was related to lower enjoyment of food even in these young children [72]. It can therefore be assumed that high levels of controlling behavior in parents might cause more difficult or conflict situations during mealtimes which potentially reduce enjoyment of food. In which way such powerful implementation might be related to food responsiveness has not been investigated so far and there is only some evidence of a potential relation between aspects of authoritarian parenting and healthy eating [73, 74], which does not correspond directly with the child’s eating behavior.

In contrast to these associations between negative parenting styles and eating behavior, our results did not reveal any relationship of positive parenting styles and eating behavior as we would have expected from a previous cross-sectional study of Goodman and colleagues [45] in young children. They had investigated 496 young children of similar age range (2.8–7.5 years). Their analyses revealed that authoritative parenting was related to less emotional overeating and less food fussiness, but in contrast to our study, analyses focused on cross-sectional data only. Besides this, other studies only focused on the relationship of positive parenting styles and feeding behavior of parents [37] and therefore evidence for the relation of positive parenting styles and the child’s eating behavior is still limited.

Although preschool age is a critical time period in the development of eating behavior, the impact of positive and negative parenting styles might be more explicit at an older age when access to food is not limited anymore.

There are several strengths and limitations in this study. First, a large sample of healthy Swiss children covering a broad range of preschool age were investigated in this study and for the first time all facets of eating behavior and the different dimensions of parenting styles were assessed which has not been done before. However, assessment techniques were limited to standardized parental questionnaires and parental responses might have been influenced by parents’ individual experiences with their children which might not correspond with an expert’s perspective. Moreover, the social desirability bias might have played a role in the assessment of negative parenting styles [51] and therefore have limited the actual magnitude of parenting styles. Furthermore, parental assessment was limited to one parent of each family and mainly mothers (only 14% of the sample were fathers). As mothers and fathers show different parenting styles [30, 75], findings might have been different if both parents had been considered in this study. In addition, each parent might base the assumptions on the child’s eating behavior depending on other expectations and experiences, and findings of this study mainly represent the maternal understanding of a child’s eating behavior. So far, fathers have rarely been assessed [76], but some research has shown that paternal feeding practices influence the child’s eating behavior too [77]. Moreover, the cross-sectional design of the study does not allow to draw any conclusion on cause-effects, and it remains unclear to which extent the child eating patterns might have influenced specific parenting styles. Furthermore, there is some evidence that similarities of parenting styles and feeding style can be expected, but they do not seem to be interchangeable [78]. Parents mostly apply different styles in eating specific and other parenting situations [79, 80]. Therefore, general parenting style and eating specific parenting style both contribute to the child’s eating behavior. It should also be kept in mind that we estimated a total of 49 (7 subscales of APQ × 7 subscales of CEBQ) associations. Based on alpha = 0.05 and assuming independent associations we would expect on average ca. 2.45 (0.05 × 49) significant effects purely by chance. Analyses revealed clearly more than 2–3 significant results, but effects were all small to medium and therefore other factors (i.e. environmental and individual aspects) might play an important role in the determination of eating behavior of a child. Furthermore, this study investigated a healthy sample of preschool children and therefore the full range of eating behavior problems might not have been represented in this sample. Further research on clinical samples need to prove to which extent parenting style can be related to eating behavior in children at preschool age.

## Conclusions

Our findings suggest that mainly negative parenting is associated with the child’s eating behavior at preschool age. Inconsistent parenting had the most consistent impact on food approach and on food avoidant behavior. Besides this, corporal punishment and powerful implementation and low monitoring were all related to mainly food approaching behavior in these young children and might be a proxy for negative family conditions which could influence eating and weight development of children in the longer term. Therefore, preventive approaches should consider negative parenting styles which seems to play a consistent role in the development of eating behavior during a critical time period of early childhood.

## Data Availability

The datasets generated and/or analyzed during the current study are not publicly available due the fact that participants were not asked at that time to provide consent on open data but are available from the corresponding author on reasonable request.

## Abbreviations

SES: Socioeconomic status
SPLASHY: Swiss Preschoolers’ Health Study
APQ: Alabama Parenting Questionnaire
CEBQ: Child Eating Behavior Questionnaire
SEM: Structural equation models SEM
